# Anticonvulsive Effect of Paeoniflorin on Experimental Febrile Seizures in Immature Rats: Possible Application for Febrile Seizures in Children

**DOI:** 10.1371/journal.pone.0042920

**Published:** 2012-08-16

**Authors:** Hitomi Hino, Hisaaki Takahashi, Yuka Suzuki, Junya Tanaka, Eiichi Ishii, Mitsumasa Fukuda

**Affiliations:** 1 Department of Pediatrics, Ehime University Graduate School of Medicine, Shitsukawa, Toon, Ehime, Japan; 2 Department of Molecular and Cellular Physiology, Ehime University Graduate School of Medicine, Shitsukawa, Toon, Ehime, Japan; 3 Ehime Proteo-Medicine Research Center, Department of Basic and Clinical Neuroscience, Ehime University, Toon, Ehime, Japan; University of Pittsburgh, United States of America

## Abstract

Febrile seizures (FS) is the most common convulsive disorder in children, but there have been no clinical and experimental studies of the possible treatment of FS with herbal medicines, which are widely used in Asian countries. Paeoniflorin (PF) is a major bioactive component of Radix Paeoniae alba, and PF-containing herbal medicines have been used for neuromuscular, neuropsychiatric, and neurodegenerative disorders. In this study, we analyzed the anticonvulsive effect of PF and Keishikashakuyaku-to (KS; a PF-containing herbal medicine) for hyperthermia-induced seizures in immature rats as a model of human FS. When immature (P5) male rats were administered PF or KS for 10 days, hyperthermia-induced seizures were significantly suppressed compared to control rats. In cultured hippocampal neurons, PF suppressed glutamate-induced elevation of intracellular Ca^2+^ ([Ca^2+^]_i_), glutamate receptor-mediated membrane depolarization, and glutamate-induced neuronal death. In addition, PF partially suppressed the elevation in [Ca^2+^]_i_ induced by activation of the metabotropic glutamate receptor 5 (mGluR5), but not that mediated by α-amino-3-hydroxy-5-methyl-4-isoxazolpropionic acid (AMPA) or N-methyl-D-aspartate (NMDA) receptors. However, PF did not affect production or release of γ-aminobutyric acid (GABA) in hippocampal neurons. These results suggest that PF or PF-containing herbal medicines exert anticonvulsive effects at least in part by preventing mGluR5-dependent [Ca^2+^]_i_ elevations. Thus, it could be a possible candidate for the treatment of FS in children.

## Introduction

FS is the most common convulsive disorder in children; more than 5% of the normal population experience at least one attack of FS, and the incidence is estimated as high as 7–14% in Japan and the Pacific islands [1]. FS can be divided into subtypes according to severity, with 30–40% of patients having a complex type with seizures that are partial, prolonged, or repeated in the same illness. Although FS does not generally induce long-term brain damage or cognitive deficits, a relationship between a history of complex FS, especially prolonged FS, during early childhood and temporal lobe epilepsy with hippocampal sclerosis has been reported [2], [3]. Therefore, it is important to prevent the recurrence of FS during childhood. Prophylactic use of antiepileptic drugs, especially phenobarbital and valproic acid, has previously been shown to reduce the recurrence of FS [4]. Unfortunately, long-term use of these drugs increases the incidence of adverse effects, such as drowsiness, hyperactivity, or learning difficulties by phenobarbital [5], and fatal liver damage by valproic acid [6]. Thus, less toxic therapeutic agents are urgently required for patients with complex FS.

Herbal medicines have been used in the treatment of several neurological disorders including epilepsy for many years [7], [8]. PF, a major component of Radix Paeoniae alba, has been used for neuromuscular blocking, cognition enhancement, and immune-regulatory function [9]–[11]. A double-blind comparative study confirmed the usefulness of PF-containing herbal medicine for adult epileptic patients [7]. However, there have been no clinical or experimental studies of herbal medicines in children with FS.

The aim of this study was to assess the efficacy of PF and a PF-containing herbal medicine against hyperthermia-induced seizures in immature rats as a model of human FS [12], [13]. Further, we investigated possible mechanisms underlying the effects of PF using cultured hippocampal neurons. We showed that PF suppressed hyperthermia-induced seizures, and that this action was associated with prevention of Ca^2+^ mobilization induced by stimulation of the metabotropic glutamate receptor (mGluR) 5. These findings suggest that PF has potential in the treatment of FS, and that the mGluR5 may be a new target for the development of new therapies for complex FS.

## Results

### Suppression of hyperthermia-induced seizures by KS and PF in immature rats

**Figure 1:**
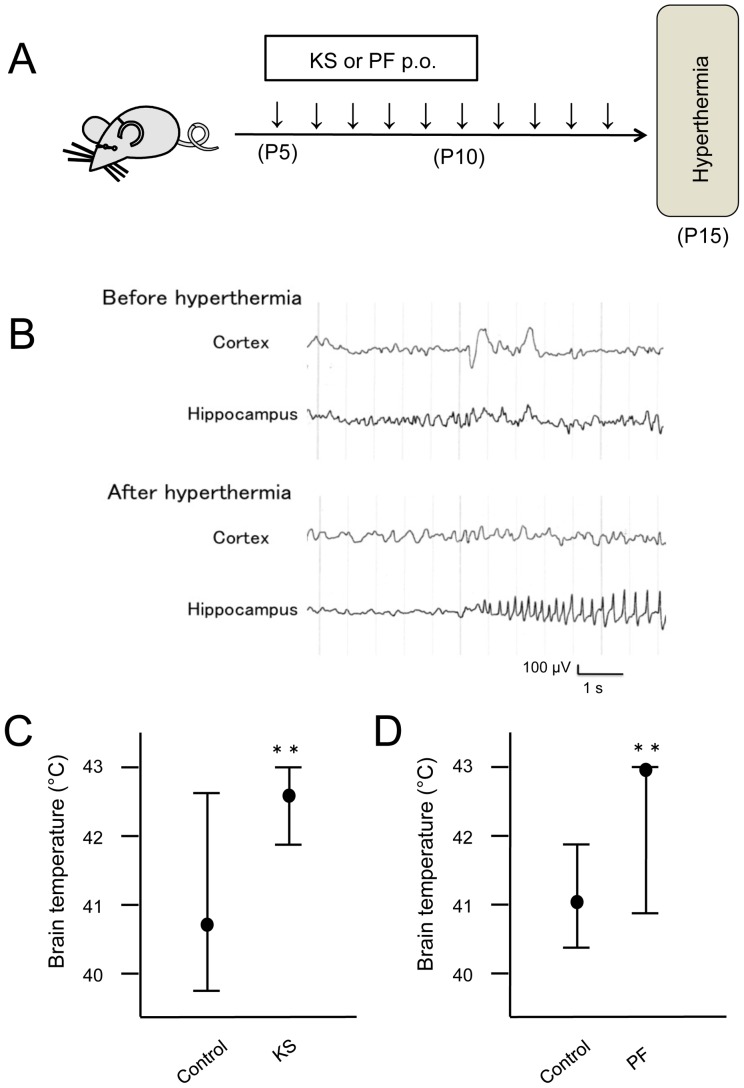
Effects of KS and PF on hyperthermia-induced seizures in immature rats. (A) Experimental schedule of drug administration. P5, P10 and P15 indicate the age of rats. (B) EEGs from cortex and hippocampus before and after hyperthermia. Before hyperthermia, EEG in both areas showed slight sustained irregular activities and a movement artifact. During hyperthermia, rhythmic ictal discharges concurrent with behavioral events were observed on hippocampal recording, and the start of this ictal discharges was defined as the onset of seizure. (C) and (D) Brain temperature at the seizure onset. The data are represented as median and range. KS, Keishikasyakuyaku-to; PF, Paeoniflorin. ** indicate P<0.01.

We evaluated the effects of PF and the PF-containing herbal medicine Keishikasyakuyaku-to (KS) on hyperthermia-induced seizures in immature rats. Oral PF, KS, or distilled water was administered for 10 days from P5, and the threshold temperature of hyperthermia-induced seizure onset was measured on P15 (Fig. 1A). Our preliminary data showed that hyperthermia induced a seizure in all rats, and that recovery was complete following the seizure. The hyperthermia-induced seizures were stereotyped, consisting of a sudden arrest of hyperthermia-induced hyperkinesias, together with facial automatism, and occasionally followed by body flexion. When the sudden immobility and facial automatism appeared at the onset of seizures, cortical EEG recordings showed moderately sustained semi-rhythmic activities or only sporadic spikes. However, deep electrodes implanted in the dorsal hippocampus revealed rhythmic ictal discharges concurrent with the onset of behavioral events (Fig. 1B). The brain temperature at the onset of the hippocampal ictal discharge was defined as the temperature at seizure onset.

As shown in Fig. 1C, the brain temperature at the onset of seizure was significantly higher in the KS group (median: 42.6°C, range: 41.9–43.0) than that in the control group (median: 40.7°C, range: 39.7–42.7°C) (P = 0.008), indicating that KS can suppress hyperthermia. The upper limit of brain temperature during hyperthermia was set at 43.0°C to avoid unphysiological hyperthermia, and one of the 6 rats in the KS group exhibited no seizure during the heating period.

We then investigated the effect of PF on seizures. The brain temperature at the onset of seizures was significantly higher in the PF group (median: 43.0°C, range: 41.9–43.0°C) than in the control group (median: 41.1°C, range: 40.5–41.8) (P = 0.001). Four of the 7 rats in the PF group did not have a seizure during the heating period (Fig. 1D). In rats of the KS and PF groups, the severity of some seizures was mild compared to the control group, with sudden immobility and facial automatism but without body flexion.

### Effect of PF on GABA concentration in cultured hippocampal neurons

**Figure 2:**
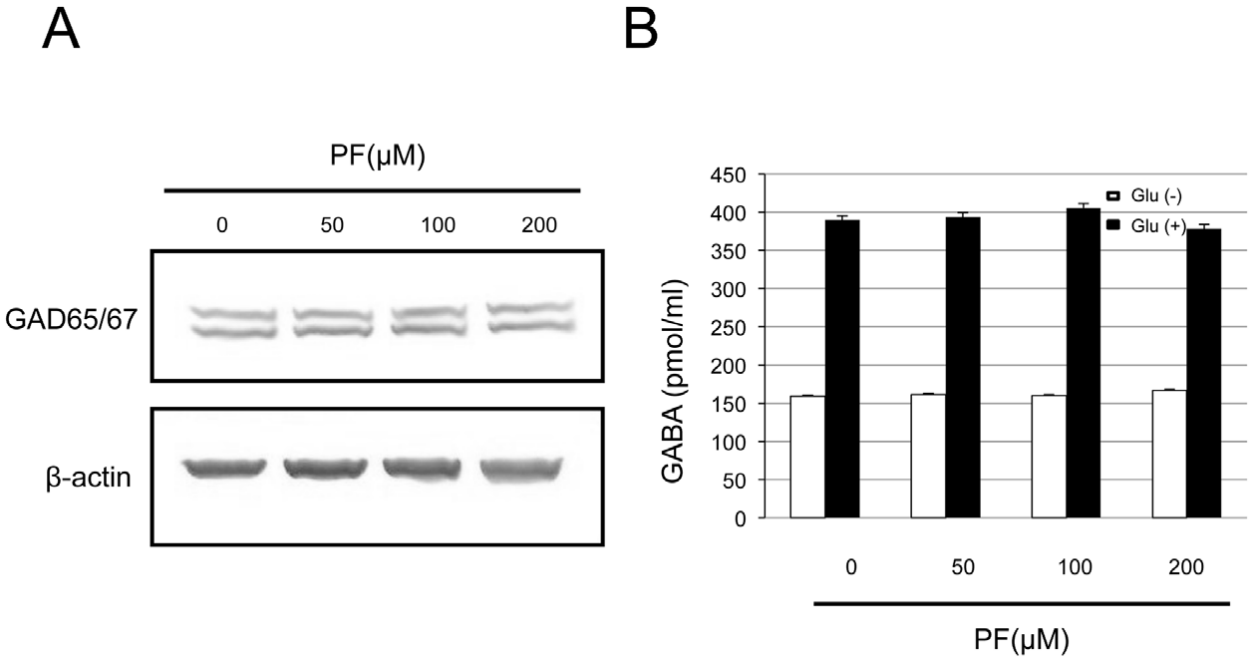
Effect of PF on the GABA production of cultured hippocampal neurons. (A) Western blot analysis of GAD65/67 protein level in cell lysates of cultured hippocampal neurons cultured in the presence of PF at the indicated concentration for 2 days. β-actin was used as a loading control. (B) Hippocampal neurons at 9 DIV were cultured in the presence of PF at the indicated concentration for 2 days. The GABA concentration was measured in the culture medium as described in Materials and Methods. The induction by L-glutamate (125 µM) was performed for 30 min in cultured hippocampal neurons. Each column represents the mean ± SE (n = 4).

We previously demonstrated that reduced activity of the GABAergic system plays the role in the pathogenesis of hyperthermia-induced seizures in immature rats [14]. We here analyzed the protein levels of the GABA synthesizing enzymes GAD65/67, to confirm whether PF suppressed hyperthermia-induced seizures via the GABAergic system. Western blotting revealed that the protein level of GAD65/67 in the cultured hippocampal neurons did not change with PF treatment (Fig. 2A). We next assessed whether PF could affect GABA release from cultured hippocampal neurons. The GABA concentration in the medium was not affected by PF treatment, whereas it was elevated 2.45 fold upon addition of glutamate, which is known to induce seizures during hyperthermia (Fig. 2B).

### Effect of PF on glutamate-induced intracellular Ca^2+^ elevation and neurotoxicity in cultured hippocampal neurons

**Figure 3:**
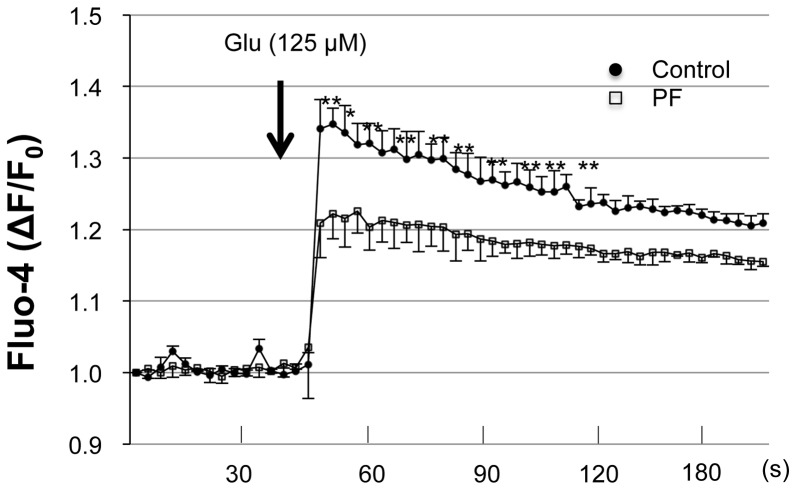
Reduction of glutamate-induced intracellular Ca^2+^ response in cultured hippocampal neurons by PF. Intracellular Ca^2+^ levels in Fluo-4-loaded cultured hippocampal neurons were monitored as the change of fluorescence intensity at 2 s intervals. Arrow indicates the time point of glutamate (125 µM) addition. Values represent mean ± SE of three independent experiments. *P<0.05 compared to control group.

The balance between glutamatergic and GABAergic neurotransmission is important in the pathophysiology of convulsive disorders and the pharmacological mechanism of anticonvulsants. As shown in Fig. 2, PF treatment did not influence the GABAergic system. Therefore, to assess the effect of PF on the excitatory system, we investigated whether PF could change the [Ca^2+^]_i_ elevation induced in hippocampal neurons by exposure to glutamate. As shown in Fig. 3, glutamate exposure induced an elevation of [Ca^2+^]_i_ in hippocampal neurons. When the neurons were pretreated with PF (200 µM) for 48 h before the glutamate exposure, the [Ca^2+^]_i_ was significantly decreased.

**Figure 4:**
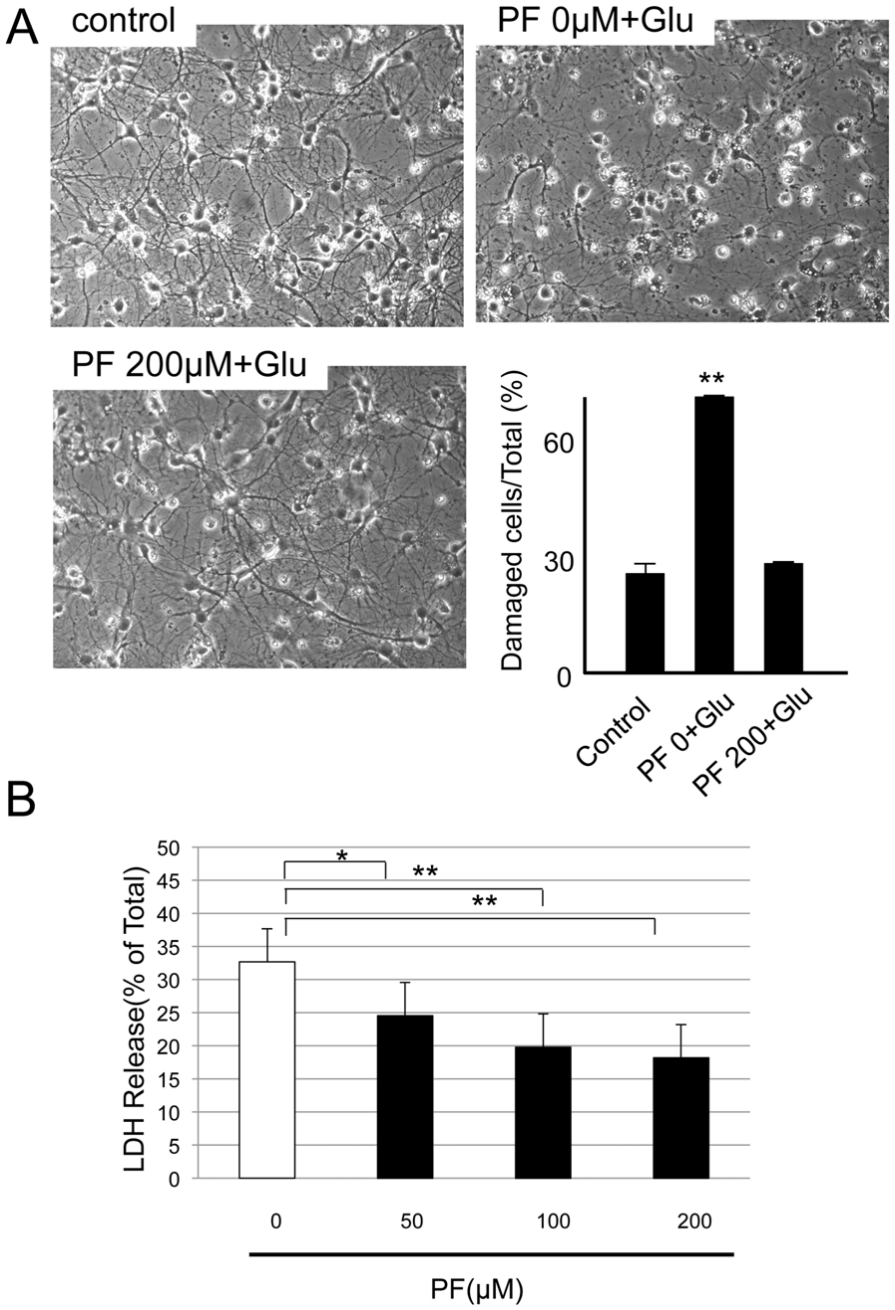
Protective effect of PF against glutamatergic neurotoxicity on cultured hippocampal neurons. Hippocampal neurons at 9 DIV were cultured in the presence of PF at indicated concentration for 2 days, treated with glutamate (500 µM) for 30 min, then the medium was replaced with culture medium followed by further culture for one day. (A) Cells were photographed under a phase contrast microscope, and quantitation of damaged hippocampal neurons with somata shrinkage after glutamatergic neurotoxicity was counted. The column indicates percentage (%) of damaged cells in total cells. Each column represents mean ± SE (n = 3). (B) Cell viability was measured by LDH assay. Each column indicates a percentage (%) of released LDH into the medium compared to total LDH. Values represent by mean ± SE (n = 4). *P<0.02, **P<0.001 compared to control group.

It is well known that aberrant elevations of [Ca^2+^]_i_ has neurotoxic effects [15]. In the current study, we observed cell damage, such as reduction of processes and shrinkage of somata, after glutamate stimulation. PF decreased this cell damage in a dose-dependent manner (Fig. 4A). We further performed a lactate dehydrogenase (LDH) cytotoxicity assay to analyze the effect of PF on cell damage. LDH release was measured to evaluate total glutamate-induced cell death. LDH release occurred during excessive glutamate stimulation, and PF decreased this LDH release in a dose-dependent manner (Fig. 4B). These results suggest that PF has an ability to suppress glutamate-induced neuronal hyperexcitability caused by aberrant elevations of [Ca^2+^]_i_.

**Figure 5:**
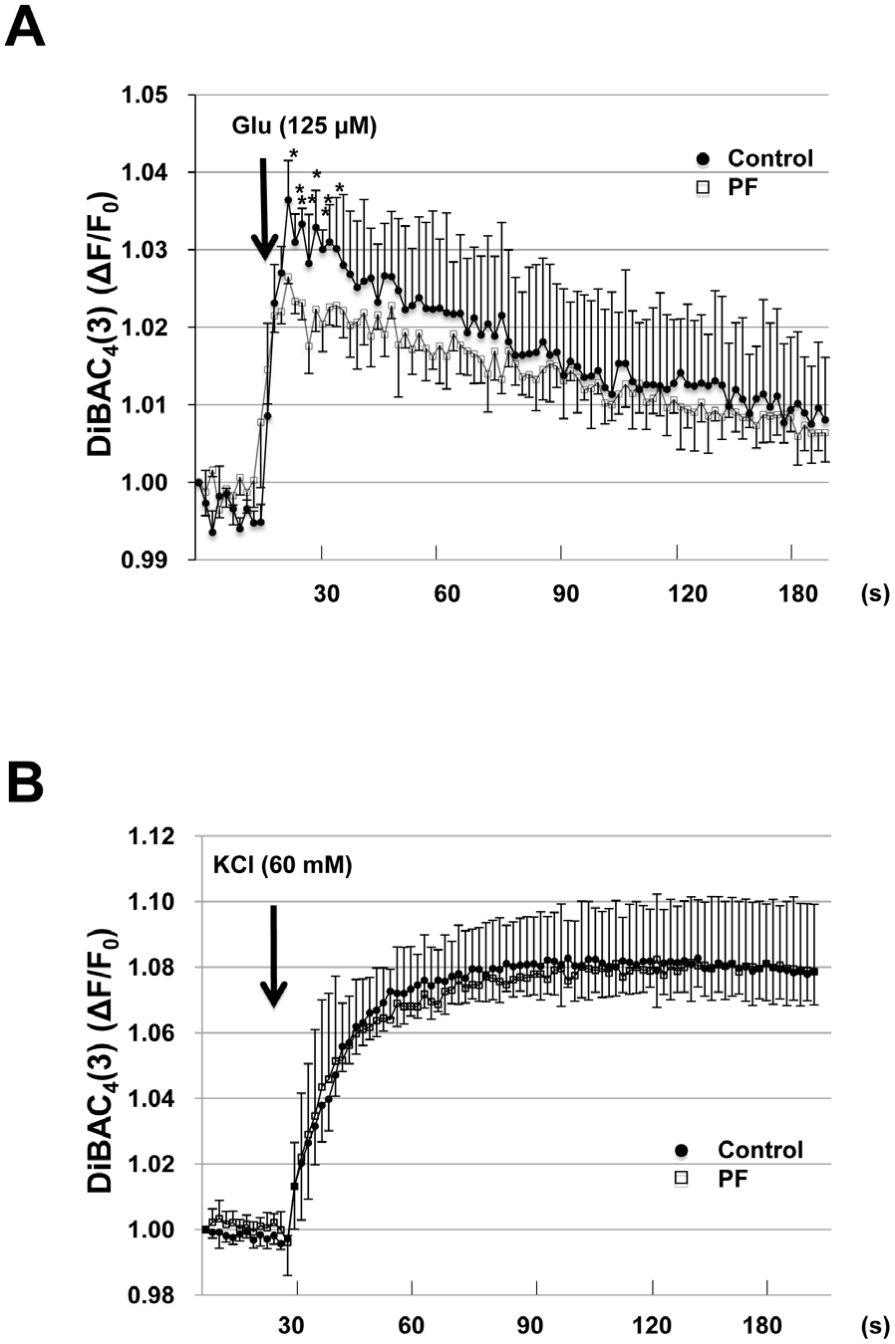
Effect of PF on the membrane depolarization of cultured hippocampal neurons. The membrane depolarization of cultured hippocampal neurons by 125 µM glutamate (A) and 60 mM KCl (B) detected as the change of fluorescence intensity of DiBAC_4_(3) at 2 s intervals. Values represent mean ± SE (n = 4). *P<0.05 compared to control group.

We further assessed changes in membrane potential using the voltage-sensitive dye DiBAC_4_(3). Glutamate caused a significant depolarization of hippocampal neurons. When neurons were treated with PF prior to glutamate stimulation, the change in membrane potential was significantly suppressed compared to control (Fig. 5A). In contrast to glutamate, KCl increases [Ca^2+^]_i_ through the opening of voltage-dependent calcium channels (VDCC). Fig. 5B shows that the same degree of depolarization was seen in PF-treated and control neurons during KCl stimulation, indicating that the effect of PF is dependent on glutamate receptors, but not on VDCCs.

### Effect of PF on elevations in intracellular Ca^2+^ concentration caused by exposure to mGluR5 agonist

**Figure 6:**
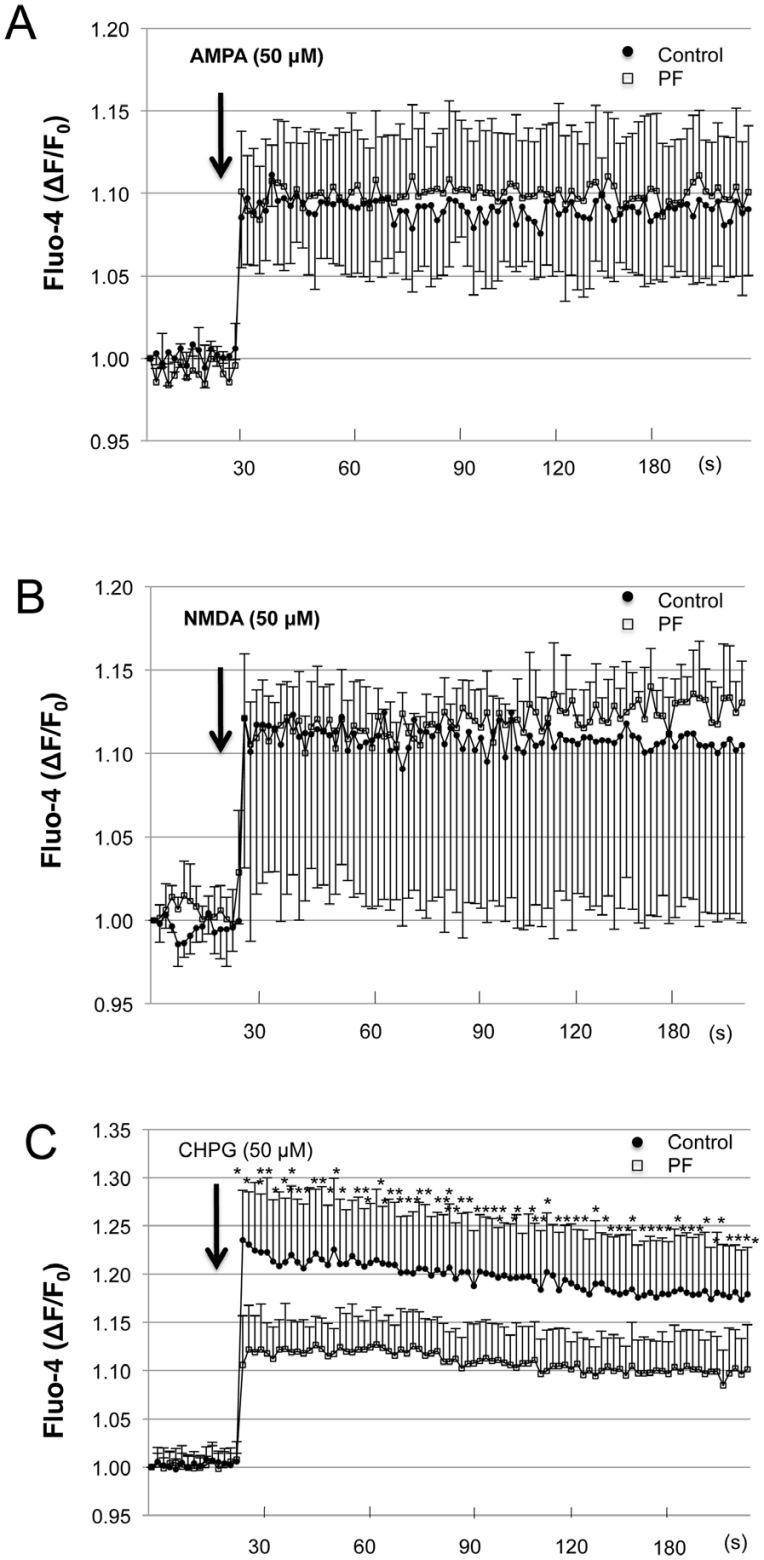
Effect of PF on intracellular Ca^2+^ response in cultured hippocampal neurons stimulated by AMPA, NMDA and CHPG. Intracellular Ca^2+^ levels in Fluo-4-loaded cultured hippocampal neurons were monitored as the change of fluorescence intensity at 2 s intervals. Arrow indicates the time point of AMPA (50 µM), NMDA (50 µM), and CHPG (50 µM) addition. Values represent mean ± SE of three independent experiments. *P<0.05 compared to control group.

We next used agonists of the NMDA receptor, AMPA receptor and mGluR5, which are the main glutamate receptors in the hippocampus, to reveal the point of action of PF [16]. PF inhibited the Ca^2+^ response induced by mGluR5 agonist, (R, S)-2-chloro-5-hydroxyphenylglycine (CHPG), but did not affect the response to NMDA or AMPA (Fig. 6).

## Discussion

Prophylactic use of antiepileptic drugs has been considered to reduce the recurrence of complex FS [4], but it can also induce several side effects, such as drowsiness, hyperactivity, or learning difficulties by phenobarbital [5] and weight gain, nausea, hair loss, or liver injury by valproic acid [6], [17]–[19]. The development of less toxic prophylactic treatments for complex FS is thus necessary to help children with FS, and we therefore analyzed the effect of a herbal medicine that has been widely used in Asian countries.

Previous studies have shown that PF-containing herbal medicines or PF metabolites inhibit experimental seizures in EL mice, a natural model of multifactorial idiopathic generalized epilepsy [20]–[22]. In addition, metabolites of PF showed not only an inhibitory effect on pentylenetetrazole (PTZ)-induced EEG power spectrum changes, but also had a protective effect on hippocampal neuronal damage in the cobalt-induced epilepsy model in adult rats [23], [24]. However, the effect of PF in infant or immature rats is still unknown.

We used rats of an immature age (P14–15) as a model of human FS [25]. Neville et al. [26] reported that the majority of FS show a focal origin and arise from the hippocampus, although FS are usually recognized as a generalized seizure type. In our immature rat model, ictal discharges during behavioral seizures originated from the hippocampus when the brain temperature was within a physiological range, consistent with previous studies [27]. Therefore, our model is thought to be suitable for the analysis of the mechanism of human FS and its prevention by less toxic drugs.

The anticonvulsive mechanisms of PF remain to be fully elucidated. Among several factors, the balance between glutamatergic and GABAergic neurotransmission may contribute to the pathogenesis of convulsive disorders. It was reported that the GABA concentration in the cerebrospinal fluid (CSF) was lower in children with complex FS than in control groups [28]. Some patients with FS-related disorders were found to have a mutation in the GABA_A_ receptor γ2 subunit [29]. The fact that the GABA_A_ receptor agonists benzodiazepine and phenobarbital are clinically effective against FS supports this possibility. We previously showed that a GABA agonist suppressed hyperthermia-induced seizures in developing rats, whereas blockade of GABA_A_ receptors or GABA synthesis enhanced them [14]. In addition, whole-cell patch clamp recordings showed that hyperthermia could suppress GABA_A_-receptor-mediated inhibition in the immature rat hippocampus [30]. These findings indicate that inadequate GABAergic function is involved in the pathogenesis of FS. On the other hand, Knight et al. [31] suggested that the CSF levels of GABA did not change in patients with first-time febrile seizures. Although further studies are needed to elucidate the GABA-dependent mechanisms of PF anticonvulsant action, we found no effects of PF treatment on GABA production or release in hippocampal neurons.

Excessive neuronal stimulation by glutamate may also be related to convulsive disorders, and is known to play an important role in the induction of experimental FS [32]. It was reported that glutamate levels in the immature rat cortex increased with the elevation in body temperature during hyperthermia-induced seizures, and the NMDA receptor blocker MK-801 inhibited the seizures [33]. Excess glutamate is toxic by increasing [Ca^2+^]_i_, free radicals and protease activity, then inducing cell death [34]. Evidence for a role of Ca^2+^ in epileptogenesis has been shown in hippocampal slices and cultured hippocampal neurons [35], [36]. Furthermore, pharmacological studies on anticonvulsant properties of Ca^2+^ antagonists in mouse have provided a direct link between Ca^2+^ channel function and susceptibility to PTZ-induced seizures [37]. Concerning the pharmacological actions of PF, it was reported that PF treatment suppressed the [Ca^2+^]_i_ elevation induced by glutamate stimulation in rat pheochromocytoma (PC12) cells [38]. However, there have been no previous reports of an involvement of Ca^2+^ signaling in the anticonvulsant actions of PF. In the current study, we showed that PF suppressed the glutamate-induced [Ca^2+^]_i_ increase and membrane depolarization in cultured hippocampal neurons, suggesting that the anticonvulsant effect of PF is Ca^2+^-dependent. This inhibiting effect of PF on [Ca^2+^]_i_ elevations may reduce the neuronal hyperexcitability induced by glutamate.

Although excessive glutamate stimulation during seizures may lead to neuronal death, hyperthermia-induced seizures themselves do not cause neuronal death in rat hippocampus [39]. However, retrospective studies have demonstrated a significant relationship between a history of prolonged FS during early childhood and TLE-HS as sequel to hippocampal neuronal death [40]. In the current study, PF suppressed cell death induced by glutamate in cultured hippocampal neurons. Continuous PF treatment in patients with complex FS might contribute to the prevention of TLE-HS.

We demonstrated that PF significantly suppressed the [Ca^2+^]_i_ elevation induced by glutamate, but not that induced by AMPA or NMDA. These results suggest that PF influences Ca^2+^ levels via mGluRs. The mGluR family comprises eight G protein-coupled receptors, which are classified into three groups (Groups I–III) according to sequence homology, effector coupling, and agonist selectivity. Group I mGluRs (mGluR1 and mGluR5) are coupled to phosphatidylinositol hydrolysis/Ca^2+^ signal transduction, and activation leads to a variety of cellular responses, including inhibition of Ca^2+^ and K^+^ currents, presynaptic modulation of synaptic transmission, and postsynaptic interaction with ionotropic glutamate receptors [41]. Group I mGluRs are especially important for the regulation of neuronal excitability. In general, activation of group I receptors enhances or facilitates the excitatory effects of glutamate by modulation of ion channel activity. Antagonists of group I mGluRs have been proposed to exhibit potential positive therapeutic effects in CNS disorders related to excessive excitatory neurotransmission, such as epilepsy, ischemia, and pain [42]. A previous study using mGluR5 knock-out mice indicated an involvement of the receptor in hippocampus-dependent learning and memory [43]. In the current study, PF treatment suppressed the [Ca^2+^]_i_ elevation caused by a selective mGluR5 agonist to about 10% of the peak in the control group. This suggests that PF might be a negative modulator of the mGluR5 and PF treatment might not cause severe side effect.

In summary, PF suppressed hyperthermia-induced seizures in immature rats, and inhibited glutamate-mediated [Ca^2+^]_i_ elevation, depolarization, and cell death via a mechanism involving the mGluR5. Our results indicate that PF may be a negative modulator of mGluR5, shedding new light on its potential in the development of new prophylactic treatments for complex FS.

## Materials and Methods

### Animals

Male Lewis rats (Charles River Laboratories Japan, Yokohama, Japan) were kept with their mothers on a 12-h light/12-h dark cycle, with free access to food and water. All experimental procedures conformed to guidelines from the Ministry of Education of Japan, and were approved by the animal experimental committee of Ehime University.

### Monitoring of electroencephalography and brain temperature

On P12, male rats were implanted with silver electroencephalography (EEG) electrodes under anesthesia with an intraperitoneal (i.p.) injection of pentobarbital sodium (25 mg/kg). Cefotaxime sodium was injected to prevent bacterial infections (500 mg/kg, i.p.). The head was fixed in a stereotaxic frame (Narishige, Tokyo, Japan), and two holes were made in the skull over the right frontal and left central regions for placement of silver cortical EEG electrodes. A stereotaxic depth EEG electrode (0.3 mm diameter; Unique Medical, Tokyo, Japan) was inserted through a central hole into the dorsal hippocampus (AP = −4.0, L = 3.0, V = −4.0 mm relative to bregma). Another hole was made over the right central cortex for placement of the needle brain temperature thermometer (Unique Medical).

### Drug administration and induction of hyperthermia-induced seizures

On P5, male Lewis rats were divided into two groups: KS (n = 7) and control (n = 8). KS (600 mg/kg; Tsumura, Tokyo, Japan) was dissolved in distilled water to a total volume of 25 µl and administered orally once a day, for 10 days starting at P5. Rats in the control group received only distilled water. On P15, rats were placed in a plastic cage and seizures were induced by moist warm air (42–43°C) while monitoring EEG activity (Fig. 1A). The brain temperature was measured at seizure onset, which was defined based on EEG and behavior. After seizure induction, rats were removed and placed on a cool surface. The upper limit of brain temperature during hyperthermia was set at 43.0°C. We carried out a similar experiment using PF. For this, rats were divided into two groups: PF (n = 8) and control (n = 8). PF (100 mg/kg; Wako, Osaka, Japan) was dissolved in distilled water to a total volume of 25 µl and orally administered daily for 10 days starting from P5. Rats in the control group received distilled water only.

After the hyperthermia-induced seizure, we applied a 10-s direct current (100 µA) to the electrodes using a current rectifier (SEN-7103; Nihon Kohden, Tokyo, Japan) to mark up the location of the electrode. Rats were anesthetized with diethyl ether (Kanto Chemical, Tokyo, Japan), and the brains were quickly removed and fixed with 4% paraformaldehyde overnight at 4°C. After dehydration in 30% sucrose for at least 24 h, the brains were sectioned coronally and stained to confirm the electrode orientation. Three rats were excluded from analysis due to inappropriate location of electrodes (one in the KS group, one in the PF group, and one in the control group).

### Hippocampal neuron culture

Rat embryos were removed from the uterus of pregnant Lewis rat at E20 and decapitated. Hippocampal tissues were carefully dissected out under the dissection microscope. Manipulations were performed under inhalation anesthesia with diethyl ether (Wako). Mechanically minced hippocampi were incubated with 0.05% trypsin/EDTA to dissociate cells for 10 min at 37°C. After the incubation, 100 µg/ml DNase I was added, and the cell suspension was triturated with pipettes. The trypsinization was quenched by fetal bovine serum. The cell suspension was filtered with a 75 µm mesh and centrifuged at 1,000 rpm. The cells were re-suspended in Neurobasal medium (Life technologies Japan, Tokyo, Japan) supplemented with B27 supplement (Life technologies Japan), 500 µM glutamine, and a penicillin/streptomycin/amphotericin B mixture (Sigma-Aldrich, St. Louis, MO, USA), and were grown for 9–11 days *in vitro* (DIV) on poly-L-lysine-coated cell culture plates.

### GABA production

Hippocampal neurons were treated with PF (0, 50, 100, 200 µM) for 48 h. To assess the effect of PF on GABA neurons, we measured GABA release in the culture supernatant of cultured hippocampal neurons before and after stimulation by glutamate (500 µM) for 30 min. GABA was detected with high performance liquid chromatography (SRL, Tokyo, Japan).

We also assessed the levels of the GABA synthesis enzyme glutamic acid decarboxylase (GAD) by western blotting. Hippocampal neurons were re-suspended in Laemmli's sample solution after treatment with PF. The lysates were electrophoresed, transferred to nitrocellulose membranes, and incubated with antibodies against β-actin (mouse monoclonal, 1∶1,000, Sigma-Aldrich) and GAD65/67 (rabbit polyclonal, 1∶1,000, Sigma-Aldrich). The blots were visualized with alkaline phosphatase-labeled secondary antibodies (Promega, Tokyo, Japan) with nitroblue tetrazolium (Sigma-Aldrich) and 5-bromo-4-chloro-3-indolyl phosphate (Sigma-Aldrich) serving as the substrates.

### LDH assay

Hippocampal neurons were exposed to a medium containing 500 µM glutamate (Wako) for 30 min. After treatment, cells were washed with culture medium or medium supplemented with PF. After 48 h, phase images of neurons were taken. To measure glutamate-induced cell death, LDH release was measured using a cytotoxicity detection kit (Roche Diagnostics, Tokyo, Japan), as previously described [44]. Briefly, hippocampal neurons at 8 DIV in 96-well plates were stimulated by glutamate (500 µM) for 30 min at 37°C. Neurons were washed in extracellular solution and incubated in cell culture medium for 16 h. Five microliters of cell lysis solution was added to cells, and the LDH activity in this sample was taken as the maximum LDH activity. After 15 min, 100 µl of the supernatant of each cell culture well was transferred to the corresponding well of a fresh 96-well plate and mixed with 100 µl of the LDH substrate. After incubation with protection from light at room temperature for 10 min, the reaction was stopped by adding 50 µl of stop buffer, and the absorbance was measured at 490 nm with a plate reader (Bio-Rad, Tokyo, Japan). The LDH release percentage was calculated by the formula: 100×(experimental culture medium background)/(maximum LDH release culture medium background). All experiments were repeated four times and the average LDH release percentage is shown in the figures.

### Ca^2+^ assay

Hippocampal neurons were seeded at a density of 50,000 cells/ml into 96-well plates. After 10 DIV, the cells were loaded with a medium supplemented with 5 µM of the Ca^2+^-sensitive fluorescent dye Fluo-4 AM (DOJINDO, Kumamoto, Japan) and 0.04% pluronic acid for 30 min at 37°C. The loading solution was then aspirated and 100 µl/well of assay buffer—Hank's balanced salt solution supplemented with 20 mM HEPES—was added. Cells were stimulated with glutamate (125 µM), NMDA (Merck, Tokyo, Japan), or AMPA (Wako), diluted in distilled H_2_O, and CHPG (Tocris Bioscience, Bristol, UK) diluted in 0.1 M NaOH. After placing both plates (cell culture and compound plate) into the FlexStation3 (Molecular Devices Japan, Tokyo, Japan), fluorescence changes were measured at 37°C. Online additions were carried out in a volume of 20 µl/well. Agonists were incubated for 5 min before addition of the agonist. The traces represent the light emission alterations of Fluo-4 (recorded between 495 nm and 518 nm), with an increase in the fluorescence ratio reflecting an elevation in cytosolic free Ca^2+^. The relative fluorescence change over time was calculated relative to the fluorescence measured at the beginning of the glutamate stimulation (ΔF/F_0_), and was expressed as relative [Ca^2+^]_i_.

### Membrane potential changes

Changes in membrane potential were measured using DiBAC_4_(3) (DOJINDO), which is a bis-barbituric acid oxonol dye with an excitation maximum at approximately 488 nm. The cells were incubated in 5 µM DiBAC_4_(3) in a HEPES-buffered solution at 37°C for 30 min prior to the fluorescence measurement. The stained cells were used for experiments without washing. Fluorescence was monitored with Flexstation3 using the excitation and emission wavelengths of 488 and 517 nm, respectively. For the experiments, background fluorescence was measured every 2 s for 2 min before addition of KCl (60 mM) or glutamate (125 µM). After adding KCl or glutamate, fluorescence was measured every 2 s for 18 min in the DiBAC_4_(3) solution.

### Statistics

To assess changes in brain temperature, GABA, and LDH, one-way ANOVA with Scheffe's multiple comparisons test were used. When data were not normally distributed, the Mann-Whitney U-test, Kruskal-Wallis test, and Dunn's post hoc test were used. For the Ca^2+^ assay and membrane potential measurements, unpaired two-tailed student's t-tests were used. SPSS version 15.0J was used for all analysis. Data were expressed as mean ± standard error (SE) or as median (range), and P<0.05 was considered to be significant.
